# Main activity trajectory clusters of unemployed people with partial work ability and cluster features

**DOI:** 10.1177/14034948231210347

**Published:** 2023-11-19

**Authors:** Joonas Poutanen, Kia Gluschkoff, Johanna Kausto, Matti Joensuu

**Affiliations:** Finnish Institute of Occupational Health, Finland

**Keywords:** Health, work ability, partial work ability, functioning

## Abstract

**Background:**

The early identification of different subgroups of individuals with partial work ability is important for the development of appropriate and effective services in order to prevent exclusion from working life and prolongation of unemployment.

**Aims:**

This study aimed to identify different main activity trajectory clusters of people with partial work ability before their participation in work ability support services and to examine sociodemographic, health, work ability and functioning features of the identified clusters.

**Methods:**

The sample consisted of clients who had participated in the Finnish Work Ability Programme during 2020–2022. Using the main activity data spanning from 2005 to 2021, optimal matching was applied to examine the similarity between the participants’ main activity trajectories. Second, using cluster analysis, participants were categorised into four main activity trajectory clusters. Finally, the sociodemographic, health, work ability and functioning features of clusters were examined.

**Results:**

A total of 643 individuals participated in the study. Four clusters were identified: (a) early-onset retirement, (b) from studies to outside the workforce, (c) from employment to unemployment and (d) long-term employment. Individuals in the ‘early-onset retirement’ cluster had the best perceived work ability and functioning. Problems relating to health, work ability, functioning and well-being were highlighted in the ‘from employment to unemployment’ cluster.

**Conclusions:**

**Unemployed individuals with partial work ability form a heterogeneous population who often have several different underlying reasons for decreased work ability. Multiple data sources are needed to identify the special characteristics and needs of the people with partial work ability.**

## Introduction

Work ability can be defined through individuals’ personal resources (health and functional capabilities, competence and values, attitudes, and motivation), the work itself and work community and leadership [1]. A person with partial work ability refers to someone who does not have full working capacity, the reasons for which may vary [2,3]. Health problems are the most common obstacle to the employment of the unemployed working-age population in Finland [4]. Long-term unemployment, financial difficulties and a low educational level explain half of low self-rated work ability [5]. It is estimated that, in Finland, there are about 65,000 unemployed individuals with partial work ability who would like to work, and it is thought that they may be capable of this in favourable circumstances [6].

As individuals with partial work ability form a heterogeneous population who often have several different underlying reasons for decreased work ability, the need for versatile and multidisciplinary services that support participation in working life are evident. However, unmet needs for health care and rehabilitation have been found to be highly prevalent among unemployed individuals with good or restricted ability to work [7]. In order to improve services, early identification of different subgroups of individuals with partial work ability and their specific needs is important.

Through registry data, it is possible to identify work disability–related subgroups and work participation trajectories based on work disability benefits, sickness absence or other work and health-related information [8,9]. For example, Lallukka et al. identified three distinctive work participation trajectories of those with mental disorders and musculoskeletal diseases [9]. In a study by Helgesson et al., work disability and unemployment trajectories of those with and without common mental disorders were identified and compared [8]. In addition to registry information, other data sources are needed to identify the special characteristics and needs of the people with partial work ability. To improve the quality and efficiency of work ability support services, it is essential to gain reliable information on clients’ needs and abilities, which can be obtained through Patient-Reported Outcome Measures (PROMs) [10]. Although the majority of PROMs are designed to assess factors such as symptom status, physical function, mental health, social function and well-being, there are also PROMs such as the Work Disability Functional Assessment Battery (WD-FAB) and the Work Ability Index (WAI) that measure work ability [11,12]. One of the most recent PROMs is the Abilitator, a self-report questionnaire on work ability and functioning, which has been developed for those in a weak labour market position [13]. Besides the psychometric studies of the Abilitator [13,14], it has been used previously in two other studies by Hult and Lappalainen and by Savinainen et al. [15,16]. One consistent finding was the significant role of physical condition in regard to self-reported with work ability of unemployed individuals.

We examined subgroups of unemployed individuals with partial work ability by combining information from national registries with the Abilitator self-reported data. The specific aims were to identify different main activity trajectories of people with partial work ability before their participation to the work ability support services, to group the trajectories into clusters based on their similarity, and to examine sociodemographic, health, work ability and functioning features of the identified trajectory clusters.

## Methods

### Setting

The Work Ability Programme (2020–2023) for unemployed people with partial work ability was implemented by the Ministry of Social Affairs and Health in conjunction with the Ministry of Employment and Economy [17]. The aim was to make individuals’ existing work ability available by supporting their work ability and functional capacity and by preventing incapacity for work [17]. The Ministry of Social Affairs and Health funded 22 government grant projects that implemented two sets of measures in 2021–2022: (a) work ability support services in social and health centres and (b) methods of supported employment [17]. The predefined target group of the programme was broad, consisting of people with partial work ability outside their working life and, for example, people with intellectual disabilities and long-term unemployment [17].

This study is part of the Finnish Work Ability Program Evaluation Study (2020–2023) conducted by the Finnish Institute of Occupational Health (FIOH) and the Finnish Institute for Health and Welfare [18]. Study participation was voluntary, and the merging of national registry and self-reported data was done based on the participants’ written research consent. The research plan was discussed in ethical committees of the FIOH and the Finnish Institute for Health and Welfare.

### Participants

The sample of this study consisted of clients (*N*=2292) who had participated in the Work Ability Programme services across Finland during 2020–2022 [18]. The total number of clients who answered the Abilitator questionnaire was 1535, of whom 670 gave their consent to link their questionnaire data with data from national registries. We excluded clients with missing data on main activity at any point of the follow-up for main activity from 2005 to 2021, which resulted in a final sample size of 643.

### Data

The registries were from Statistics Finland, the Social Insurance Institution of Finland, the Finnish Centre for Pensions and the Finnish Institute for Health and Welfare. Data on year of birth, sex, main activity at the end of the year (e.g. employed, unemployed, studying or retired from 2005 to 2021), gross income (in 2020) and education (in 2022) were drawn from Statistics Finland. Data on the number of months receiving income support were drawn from the registers of the Social Insurance Institution of Finland. The number of days (before the end of 2021) that the client had no information in the earnings and accrual register (i.e. the number of days the client had not received wages or social benefits for which pension accrues), reflecting time spent outside the workforce, was obtained from the Finnish Centre for Pensions. Data on the number of health-care visits (any diagnosis) in 2020–2022 and on mental disorder and musculoskeletal diagnoses in 2020–2022 were drawn from the Care Register for Health Care, managed by the Finnish Institute for Health and Welfare.

Self-reported work ability and functioning data were measured with the Abilitator. The Abilitator was developed by the FIOH, and its psychometric properties have been evaluated in three studies showing adequate reliability and validity [13–15]. The Abilitator contains a total of 84 questions in the following sections: personal details (e.g. age, gender), well-being (e.g. general functioning, perceived work ability), inclusion (social functioning and social interaction), mind (mental functioning), everyday life (coping with everyday activities), skills (e.g. cognitive functioning, competence), body (physical functioning), background information (e.g. educational background) and work and the future (e.g. employment situation, desired changes) [13]. The scales and interpretation of variables that were used in this study are presented in Table I. The participants answered the Abilitator questionnaire when entering the Work Ability Programme services.

**Table I.** The Abilitator variables that were utilised in the study. Sections C, D, E, F and G consists of several questions which are measured mostly with a 1–5 Likert scale.

Variable	Scale and interpretation
(B1) How satisfied are you with your life at this moment? Assess your general satisfaction with life.	5 Very satisfied 4 Fairly satisfied 3 Neither satisfied nor dissatisfied 2 Fairly dissatisfied 1 Very dissatisfied
(B2) In your opinion, how is your health currently? Assess your health as a whole.	5 Good 4 Fairly good 3 Average 2 Fairly poor 1 Poor
(B3) How well do you cope with your everyday activities and tasks? Choose the number that best matches your situation. Assess your everyday life in general and how you cope with it.	0 1 2 3 4 5 6 7 8 9 10 0=I cope very poorly 10=I cope very well
(B4) Let’s assume that your work ability would receive a score of 10 points at its best. What score would you give your current work ability? If you do not currently work, give your assessment in relation to your last job or the demands of your occupation. If you have no profession, assess your situation in relation to the work you would like to do.	0 1 2 3 4 5 6 7 8 9 10 0=Completely unable to work 10=Work ability at its best
(B5) How do you feel in relation to working life at the moment? Choose the number that best matches your situation.	0 1 2 3 4 5 6 7 8 9 10 0=Work life or employment does not currently apply to me. 1–3=I don’t have a job. I’m poorly equipped for working life. I need support in order to obtain employment. 4–5=I don’t have a job, but I am equipped for working life. I may need support in order to obtain employment. 6–8=I have a job. I am equipped for working life. I may, however, need support in order to stay in employment. 9–10=I have a job. I am well equipped to continue in employment.
(C) Inclusion (social functioning and social interaction)	0%–23%=poor situation, 25%–48%=fairly poor situation, 50%–73%=fairly good situation, 75%–100%=good situation
(D) Mind (mental functioning)	0–22%=poor situation, 25–56%=possible challenges, 58–100%=good situation
(E) Everyday life (coping with everyday activities)	0%–23%=poor situation, 25%–48%=fdairly poor situation, 50%–73%=fairly good situation, 75%–100%=good situation
(F) Skills (e.g. cognitive functioning, competence)	0%–48%=poor situation, 50%–73%=possible challenges, 75%–100%=good situation
(G) Body (physical functioning)	0%–30%=poor situation, 40%–80%=possible challenges, 90%–100%=good situation
Overall situation	0%–100%
(H2) Does the total income of your household cover your costs:	6 Very easily 5 Easily 4 Fairly easily 3 Fairly poorly 2 Poorly 1 Very poorly
(I2) How long has your current period of unemployment lasted? If you are in rehabilitation etc., consider the duration of your unemployment before this.	1 Less than a year 2 1–2 years 3 3–4 years 4 5–7 years 5 8–10 years 6 More than 10 years
(I14) Which areas of your life do you wish to change? You may choose more than one option.	1 My work or employment situation 2 My competence and professional skills 3 My financial situation 4 My health 5 My sleep and body clock rhythm 6 My diet 7 My physical fitness 8 Management of everyday life 9 My emotional well-being 10 My personal relationships 11 My hobbies and general ability to participate 12 My use of alcohol, drugs or other addictions 13 I don’t know 14 I feel no need for improvements

The national registry and self-reported work ability and functioning data were linked based on the unique individual numbers of the participants.

### Statistical analysis

To assess sampling bias risk, we compared selected Abilitator results (age, gender, work ability, functioning and unemployment duration) between study participants and those who did not consent to link their questionnaire data with national registers. The main analysis involved three steps. First, we used optimal matching with the dynamic Hamming distance algorithm to analyse participants’ main activity trajectories, which were sequences of main activity states (employed, unemployed, <15 years of age, student, retired or other) [19]. The method compares the degree of dissimilarity between each pair of main activity sequences and calculates distances between individuals based on their sequence [20]. In this kind of matching process, two individuals who have been unemployed at different times during the follow-up are considered more distant from each other than individuals who have been unemployed synchronously. When calculating the distances, the algorithm assigns a cost for states that do not match (i.e. if a state for a specific year during the follow-up is coded as ‘employed’ for one person and ‘unemployed’ for the other). For non-matching states, a substitution is required for the states to match, changing the status, for example, from ‘employed’ to ‘unemployed’. The substitution costs are time-varying because they are derived from transition probabilities between the states at each time point. In contrast to some other sequence analysis methods, the method that was used in this study does not ‘warp’ time because only substitutions, and not insertions or deletions, are used. Therefore, the method can be applied only to sequences of the same length. The method is typically used for comparing sequences when the focus is on the timing of the transitions between the states (e.g. whether an individual becomes unemployed at the beginning or end of the follow-up) and the duration of stay in each state (e.g. the length of an unemployment spell).

As the second step, after using optimal matching to calculate the distances between individuals, cluster analysis with a partition around medoids algorithm was used on the distances to determine individuals with similar main activity sequences [21]. The ideal cluster count was determined using the average silhouette width index.

Third, we examined sociodemographic, health, work ability and functioning features of trajectory clusters using analysis of variance for continuous variables and Pearson’s chi-square or Fisher’s exact tests for categorical variables. These analyses were conducted using R v4.0.5 (R Foundation for Statistical Computing, Vienna, Austria) [22].

## Results

Overall, the differences in the Abilitator results were small between the study sample and those who did not give their consent to link their questionnaire data with data from national registers. Those who gave their consent to link the data were slightly older, had slightly poorer work ability and functioning, and had been unemployed for a longer period of time. Overall, the magnitudes of the differences were small.

**Figure 1.**
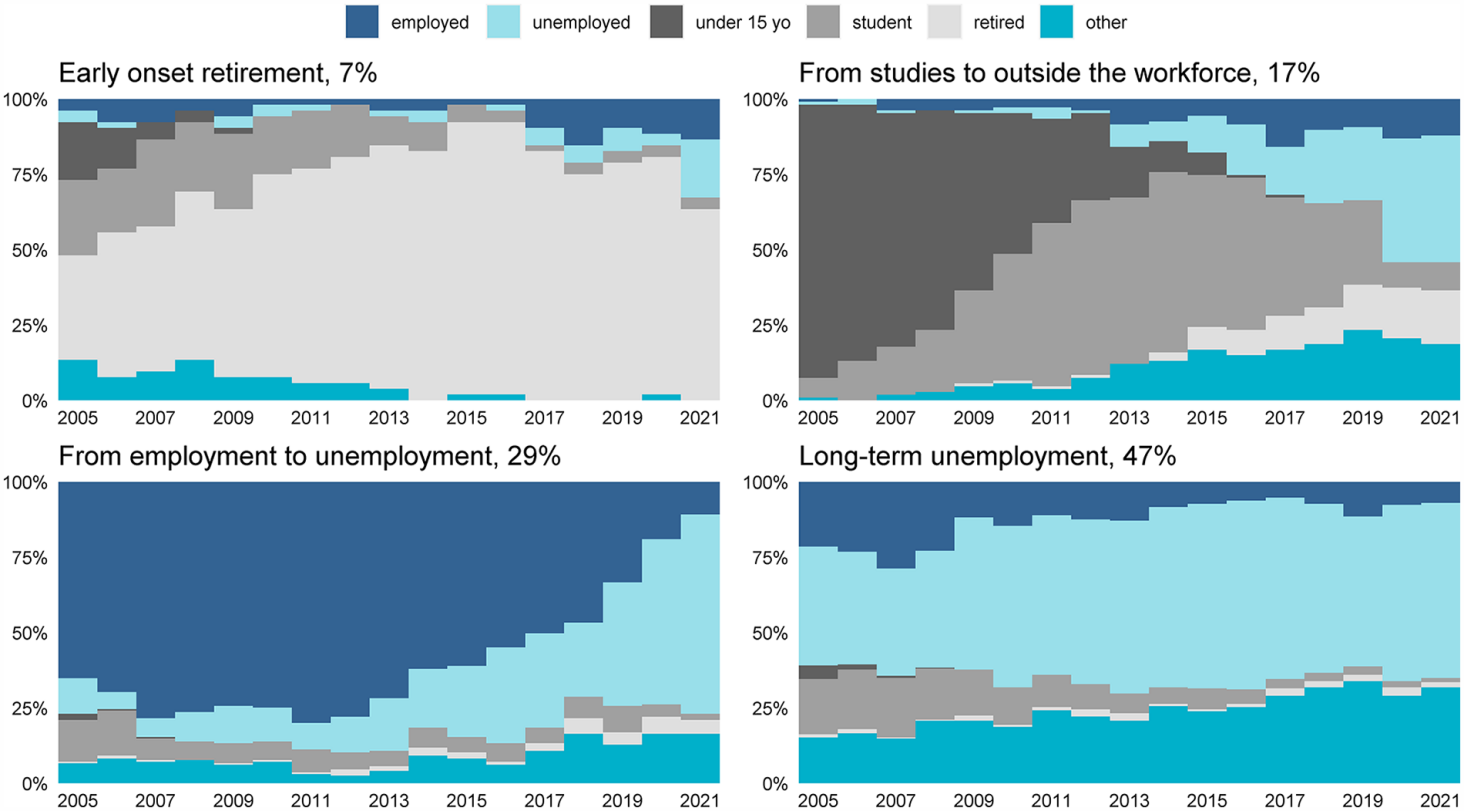
A chronogram (i.e. a state proportion plot) describing the distributions of main activity states during the follow-up in each cluster.

Based on the results of optimal matching and cluster analysis, a four-cluster solution was selected for further analysis. Figure 1 presents chronograms (i.e. state proportion plots) for each cluster, showing the distribution of the various states at each year of the follow-up. The clusters were named according to their characteristic features: (a) early-onset retirement (*n*=52; 7%), (b) from studies to outside the workforce (*n*=107; 17%), (c) from employment to unemployment (*n*=195; 29%) and (d) long-term employment (*n*=289; 47%).

Table II presents the sociodemographic, health, work ability and functioning features according to the national registries and self-reported data, and statistical differences between the clusters. Overall, the clusters differed the most in terms of age, mean gross income, life satisfaction, health, work ability, relation to working life, unemployment duration, body and overall scores.

**Table II.** National registry and self-reported work ability and functioning data.

Variable		Overall, *N*=643	Early-onset retirement, *N*=52	From studies to outside the workforce, *N*=107	From employment to unemployment, *N*=195	Long-term unemployment, *N*=289	*p*-Value	Effect size	Valid *N*
Registry data	Age, 2021	43.57 (12.00)	38.81 (9.10)	26.27 (4.88)	49.09 (9.13)	47.11 (9.65)	<0.001	Large	643
	Gender						0.031	Small	643
	Male	286 (44%)	30 (58%)	51 (48%)	72 (37%)	133 (46%)			643
	Female	357 (56%)	22 (42%)	56 (52%)	123 (63%)	156 (54%)			643
	Education, 2022						<0.001	Small	643
	Tertiary	96 (15%)	0 (0%)	10 (9.3%)	46 (24%)	40 (14%)			
	Primary	159 (25%)	23 (44%)	26 (24%)	22 (11%)	88 (30%)			
	Secondary	388 (60%)	29 (56%)	71 (66%)	127 (65%)	161 (56%)			
	Health-care visits, 2020–2022	73.98 (71.74)	47.42 (57.86)	62.26 (50.22)	91.59 (93.87)	71.22 (60.17)	<0.001	Small	643
	Mental health diagnosis	319 (50%)	8 (15%)	53 (50%)	112 (57%)	146 (51%)	<0.001	Small	643
	Musculoskeletal diagnosis	319 (50%)	11 (21%)	29 (27%)	124 (64%)	155 (54%)	<0.001	Small	643
	Income support months, 2020–2022	12.21 (14.29)	0.54 (2.67)	9.74 (13.12)	10.03 (12.89)	16.70 (15.11)	<0.001	Medium	643
	Days outside the workforce	1600.58 (1634.12)	1842.60 (2127.03)	1087.93 (1282.68)	985.48 (1017.84)	2222.98 (1823.05)	<0.001	Medium	541
	Unknown		22	33		47			
	Mean gross income in 2020	11,604.07 (6108.00)	10,962.01 (2389.00)	8889.47 (3979.40)	15,337.54 (8199.62)	10,205.53 (4101.68)	<0.001	Large	643
Self-reported data	Life satisfaction (B1)	3.10 (1.08)	4.42 (0.78)	3.36 (1.08)	2.81 (0.97)	2.96 (1.01)	<0.001	Large	642
	Health (B2)	2.78 (1.15)	4.27 (0.93)	3.29 (1.27)	2.43 (0.91)	2.57 (1.00)	<0.001	Large	640
	General functioning (B3)	5.76 (2.35)	8.31 (1.98)	6.15 (2.31)	5.12 (2.21)	5.58 (2.21)	<0.001	Medium	643
	Work ability (B4)	4.46 (2.70)	8.02 (2.22)	5.27 (2.87)	3.49 (2.18)	4.17 (2.43)	<0.001	Large	643
	Relation to working life (B5)	3.95 (2.01)	6.00 (2.48)	4.48 (2.16)	3.66 (1.75)	3.52 (1.69)	<0.001	Large	532
	Unknown			15	41	52			
	Sufficiency of income (H2)	3.15 (1.46)	4.79 (1.29)	3.74 (1.36)	2.70 (1.34)	2.94 (1.32)	<0.001	Medium	642
	Unemployment duration (I2)	3.43 (1.72)	4.73 (1.85)	2.51 (1.34)	2.83 (1.43)	4.06 (1.73)	<0.001	Large	542
	Inclusion (C)	60.42 (20.72)	82.60 (16.32)	61.59 (21.83)	57.26 (19.25)	58.03 (19.54)	<0.001	Medium	633
	Mind (D)	56.90 (17.40)	72.46 (17.54)	58.89 (17.35)	54.06 (16.98)	55.25 (16.17)	<0.001	Medium	634
	Everyday life (E)	70.68 (18.43)	79.48 (15.56)	72.41 (18.24)	68.23 (18.22)	70.08 (18.67)	<0.001	Small	632
	Skills (F)	61.96 (17.67)	77.69 (15.45)	65.51 (14.98)	61.11 (18.03)	58.30 (16.96)	<0.001	Small	627
	Body (G)	57.50 (26.83)	82.69 (17.05)	70.86 (21.80)	50.58 (26.66)	52.56 (25.73)	<0.001	Large	631
	Overall	61.61 (16.05)	78.96 (12.88)	66.05 (15.39)	58.01 (15.10)	59.00 (14.95)	<0.001	Large	
	Unknown			7	14	22			
	Desired changes (I14)								
	Employment	346 (54%)	29 (56%)	71 (66%)	110 (56%)	136 (47%)	0.005	Small	643
	Competence and skills	203 (32%)	16 (31%)	47 (44%)	64 (33%)	76 (26%)	0.010	Small	643
	Financial situation	398 (62%)	25 (48%)	60 (56%)	129 (66%)	184 (64%)	0.054	Small	643
	Health	441 (69%)	14 (27%)	62 (58%)	158 (81%)	207 (72%)	<0.001	Medium	643
	Sleep	277 (43%)	16 (31%)	43 (40%)	91 (47%)	127 (44%)	0.2	–	643
	Diet	207 (32%)	16 (31%)	42 (39%)	58 (30%)	91 (31%)	0.4	–	643
	Physical fitness	350 (54%)	20 (38%)	50 (47%)	114 (58%)	166 (57%)	0.017	Small	643
	Everyday life	179 (28%)	14 (27%)	40 (37%)	59 (30%)	66 (23%)	0.029	Small	643
	Emotional well-being	350 (54%)	14 (27%)	59 (55%)	118 (61%)	159 (55%)	<0.001	Small	643
	Relationships	157 (24%)	12 (23%)	24 (22%)	48 (25%)	73 (25%)	>0.9	–	643
	Hobbies	180 (28%)	15 (29%)	35 (33%)	57 (29%)	73 (25%)	0.5	–	643

*p*-Values from analysis of variance (ANOVA) for continuous variables and from Pearson’s chi-square test or Fisher’s exact test for categorical variables. For ANOVA, we calculated eta squared (η^2^) for effect size and used the following cut-off values: η^2^=0.01, small effect; η^2^=0.06, medium effect; and η^2^=0.14, large effect. For chi-square and Fisher’s exact tests, we calculated Cramer’s V for effect size and used the following cut-off values: V=0.1, small effect; V=0.3, medium effect; V=0.5, large effect. Effect sizes below ‘small’ are not shown.

Individuals in the ‘early-onset retirement’ cluster had the longest self-reported duration of unemployment, and the proportion of primary-level education was the largest among the clusters. The number of months receiving income support was lowest, and these individuals had the fewest health-care visits and mental disorder and musculoskeletal diagnoses. Overall, the individuals in the cluster had the best Abilitator scores, demonstrating better perceived work ability and functioning compared to other clusters. The most-often desired changes were related to work or employment situation (56%), financial situation (48%) and physical fitness (38%).

Individuals in the ‘from studies to outside the workforce’ cluster were, on average, the youngest and had the lowest income in 2020. Compared to the ‘from employment to unemployment’ and ‘long-term unemployment’ clusters, the proportion of those who had musculoskeletal disorders was smaller. Half of the individuals in this cluster had a diagnosis of a mental disorder. Mental health challenges were also reflected in the Abilitator’s ‘mind’ section scores. However, the Abilitator scores were the second best behind the ‘early-onset retirement’ cluster. The most-often desired changes were related to work or employment situation (66%), financial situation (56%) and health (58%).

Individuals in the ‘from employment to unemployment’ cluster were the oldest and had the highest level of education compared to the other clusters. They also had the shortest unemployment duration in 2020–2022 and highest income in 2020 according to registry history. Problems relating to health, work ability, functioning and well-being were highlighted in the cluster; the individuals had the most mental disorder and musculoskeletal diagnoses and health-care visits. They also had the lowest self-rated life satisfaction, health, general functioning and work ability scores, and the lowest ‘inclusion’, ‘mind’, ‘everyday life’, ‘body’ and ‘overall’ scores as well. Members of this cluster were also the most dissatisfied with the sufficiency of income. The most-often desired changes were related to health (81%), financial situation (66%) and emotional well-being (61%).

Individuals in the ‘long-term unemployment’ cluster had the longest duration away from working life according to the registry data. Participants also had the most income support months and lowest relation to working life and ‘skills’ scores. Overall, problems relating to health, work ability, functioning and well-being were the second most significant behind the ‘from employment to unemployment’ cluster. The most-often desired changes were related to health (72%), financial situation (64%) and physical fitness (57%).

## Discussion

In this study, four clusters of unemployed people with partial work ability were identified with differentiating sociodemographic, health, work ability and functioning features. Previously, the heterogeneity of the unemployed people and work participation trajectories has been studied by using both register and self-reported data [4,8,9]. However, we were able to examine the heterogeneity of unemployed people with partial work ability in parallel by using both register and self-reported information. Clusters of unemployed people with partial work ability can be identified from registry data, and they show different characteristics in self-reported data on work ability and functioning. As people with partial work ability form a heterogeneous population with multifaceted challenges, identifying different subgroups is important for the development of appropriate and effective services. According to the findings of the study, it is possible to form case descriptions and suggestions of service pathways for the four identified clusters.

It can be assumed that the members in the ‘early-onset retirement’ cluster may feature individuals with developmental disabilities. The assumption is supported by the large proportion of pension recipients, as well as participation in the employment interventions supported by the Work Ability Programme, which were targeted, for example, at people with developmental disabilities. The individuals in this cluster had the fewest health-care visits and mental disorder and musculoskeletal diagnoses and had the best self-rated work ability and functioning scores among the identified clusters. In a previous study by Boland et al., it was also observed that individuals with intellectual disability rated their general health and quality of life highly [23]. This may be explained by the fact that the disability of these individuals is congenital so, their situation might be more stabilised compared to individuals whose health status has suddenly changed. Due to relatively good health, work ability and functioning situation and orientation towards working life, services where the goal is a quick transition to the open labour market, such as Supported Employment (SE) and its evidence-based model Individual Placement and Support (IPS), are suitable. These vocational rehabilitation approaches have been found to be effective in improving vocational and non-vocational outcomes and cost-effectiveness as well compared with other vocational rehabilitation approaches or usual care [24,25].

The cluster ‘from studies to outside workforce’ could be described as marginalised young people who have not been employed due to various underlying challenges. The situations of individuals in this cluster and the reasons for being excluded from working life may be more unclear, and there can be several explanatory factors in the background. Previous research has discovered that young adults at risk of work disability often have multifactorial challenges in the background such as childhood adversity, psychological symptoms, alcohol use and reading and writing difficulties [26,27]. The results of this study partly confirmed these previous findings, as challenges related especially to mental health were emphasised in the cluster’s register and self-reported data. These individuals may need services that include a comprehensive assessment of the situation and work ability, with the focus especially on psychological and social factors, while orientating towards working life [26].

Musculoskeletal and mental disorders are known as the key reasons to exit paid employment due to disability [9], and these factors were also highlighted in the ‘from employment to unemployment’ cluster. Individuals in this cluster are the ones who had a working career in the past, but due to health problems or illness, they have moved outside of working life. Vocational rehabilitation interventions which focus on diminishing the limitations and restrictions identified during the assessment, for example increasing fitness, work conditioning, ameliorating anxiety or depression, building confidence and training in the management of stress, can be appropriate for the participants in the cluster [28].

Individuals in ‘long-term unemployment’ had the second-lowest self-rated work ability and functioning scores overall. This is in line with previous research which has shown that prolonged unemployment is associated with decreased work ability [29]. Members of this cluster were also the furthest away from working life based on the data. The lowest scores in the ‘skills’ section also reflect that, as a result of prolonged unemployment, these people do not feel that their competencies are sufficient for the demands of working life. It is known that low educational level is one important factor that can negatively affect participation in working life and work ability [5]. In the ‘long-term unemployment’ cluster, the proportion of those with primary-level education was the second largest behind the ‘early-onset retirement’ cluster. Interventions for individuals in this cluster may vary between those previously mentioned, but the most essential feature of the service is to identify the person’s existing work ability and to orient them towards education and working life.

The strength of this study was that it was possible to combine register and self-reported data, which enabled a multidimensional description of the target group. The other advantages were the use of multiple national registries and the use of the self-report questionnaire on work ability and functioning, which is aimed specifically at the population in a weak labour market position. The study also had limitations. The sample consisted of voluntary participants, not a random sample of the total participants of the Work Ability Programme, which can lead to selection bias. The sample was also relatively small, especially the ‘early-onset retirement’ cluster, compared to the total number of clients in the Work Ability Programme. However, no remarkable differences were observed in the Abilitator results of the study sample compared to those who did not participate in the study. Nevertheless, the results need to be replicated in larger samples in the future. Due to the nature of the main activity classification, we were unable to identify individuals who were on sickness absence. Including sickness absence as an additional state could be a valuable avenue for future research.

## Conclusions

Four clusters of unemployed people with partial work ability were identified, with specific sociodemographic, health, work ability and functioning features according to the registries and self-reported data. The identified specific features and needs of the individuals with partial work ability, as well as the suggestions of service pathways, can be applied in the development and targeting of work ability support services to different target groups. The early identification of different subgroups can aid the development of appropriate and effective services in order to prevent exclusion from working life and prolongation of unemployment.
